# Synthetic Diastereomers of the Fungal Cycloheptapeptide Paraisariamide A Retain Ternatin-Like Biological Activity

**DOI:** 10.1021/acsmedchemlett.6c00180

**Published:** 2026-07-01

**Authors:** Sophia E. Bonar, Daphne R. Mattos, Takumi Arai, Yui Tagami, Dean A. Buch, Richard M. Tehan, Kerry L. McPhail, Shinya Oishi, Jane E. Ishmael

**Affiliations:** ‡ Department of Pharmaceutical Sciences, College of Pharmacy, 2694 Oregon State University, Corvallis, Oregon 97331, United States; § Laboratory of Medicinal Chemistry, 12916 Kyoto Pharmaceutical University, Kyoto 607-8412, Japan; ∥ Department of Medicinal Chemistry, College of Pharmacy, 7060 University of Utah, Salt Lake City, Utah 84103, United States

**Keywords:** protein synthesis inhibitor, SUnSET assay, didemnin B, ternatin peptide, fungal pathogen

## Abstract

Paraisariamides (Psds) are a family of fungal N-methylated cyclic heptapeptides with structural similarity to the translation eukaryotic elongation factor 1A inhibitor ternatin. Initial characterization of the eight known Psds A–H revealed a complex biological activity profile; Psd A and Psds E–H showed differential cytotoxicity to HCT116 colon cancer cells, whereas Psds B–D were inactive. Here, we report that Psd A is a weak inhibitor of total protein synthesis and that three synthetic diastereomers of Psd A retain comparable pharmacological activity. Psd A and all structural variants decreased the level of expression of extracellular *Gaussia* luciferase in a phenotypic assay that senses endoplasmic reticulum stress in living cells. Inhibition of cellular secretion was fully reversible; however, the three Psd A diastereomers were all eventually cytotoxic to HCT116 colon cancer cells. The stereochemistry of Psd A, a weak cytotoxin relative to Psds E–H, is anticipated to inform future structure–activity relationship studies of these ternatin-like molecules.

## Graphical abstract

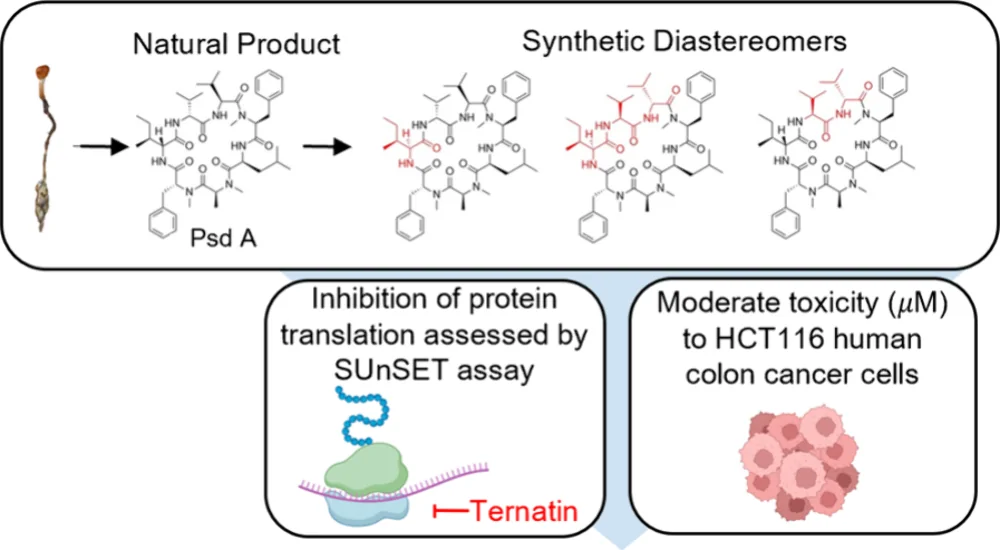

Paraisariamides (Psds) are a recently described family of N-methylated cyclic heptapeptide natural products produced by several *Paraisaria* species of insect-pathogenic fungi.^1^ To date, the absolute structures of eight paraisariamides (Psds A–H) have been elucidated from *Paraisaria* fungi collected in North America and confirmed by total synthesis of key members of the series.^1^ Psds A–D were isolated from axenic laboratory cultures of *Paraisaria cascadensis*, which infects the great grig (*Cyphoderris monstrosa*), and Psds E–H were identified from cultures of the longhorn beetle larva pathogen *Paraisaria insignis*.^1,2^ While the Psds occur as complex mixtures in each of the host organisms,^1,2^ comparative analysis of the pure natural and synthetic products revealed a complex, cell-type specific biological activity profile.^1^ Human HCT116 colon cancer cells were sensitive to a subset of the Psds in standard end point viability assays, whereas human U87-MG glioblastoma cells were fully resistant.^1^ Further pharmacological characterization of the Psds revealed concentration-dependent decreases in viability only in HCT116 cells exposed to Psd A (**1**) and Psds E–H, while Psds B–D were essentially inactive.^1^ The five cytotoxic Psds also displayed variable activity against HCT116 cells, ranging from low nanomolar potency for Psds E and F to micromolar potency for Psd A (**1**).^1^ Based on structural similarity of the Psds to the fungal heptapeptide ternatin,^3^ an inhibitor of translation eukaryotic elongation factor 1A (eEF1A),^4^ we hypothesized that the cytotoxic potential of the Psds may be attributed to a similar inhibition of protein translation at the level of the ribosome. The most cytotoxic Psds (E–H) were, in turn, confirmed to induce a concentration-dependent loss of protein synthesis after metabolic labeling of proliferating HCT116 cells with l-azidohomoalanine (l-AHA), in methionine-free culture medium, and detection of nascent protein with tetramethylrhodamine (TAMRA)-phenyl azide.^1^

Our preliminary pharmacological characterization of the Psd series was enabled by synthetic chemistry, in that total synthesis of paraisariamide A (**1**) was critical for final assignment of the absolute configuration of the natural product^1^ (Figure 1). Although the advanced Marfey’s method for configurational analysis of pure **1** from *P. cascadensis* confirmed the presence of all seven component amino acids, including d- and l-valine, this approach was unable to distinguish the relative position of d- versus l-valine in the molecule.^1^ Thus, both synthetic products were targeted using solid-phase peptide synthesis to yield paraisariamide A (**1**) with d-Val^6^/l-Val^7^ and [l-Val^6^, d-Val^7^]-paraisariamide A (**4**). Comparative analysis of these two molecules allowed formal assignment of the authentic natural product as **1**.^1^ In the process of synthesizing peptides **1** and **4**, two additional cycloheptapeptides were generated during the final synthetic step: [l-lle^5^]-paraisariamide A (**2**) and [l-lle^5^, l-Val^6^, d-Val^7^]-paraisariamide A (**3**) (Scheme S1). These presumed unnatural Psds were synthesized as side products arising from racemization of the α position of d-*allo*-isoleucine (d-*allo*-Ile) during macrocyclization between d-*allo*-Ile^5^ and Val^6^. The presence of l-Ile^5^ in heptapeptides **2** and **3** was verified by comparative analysis with the products obtained via an alternative synthetic route including macrocyclization of l-Ile^5^-containing linear precursors between l-Leu^2^ and l-MeAla^3^ (Scheme S2). Thus, in addition to the target products **1** and **4** containing d-*allo*-Ile^5^, racemization yielded two additional products (**2** and **3**) containing l-Ile^5^ (Figure 1 and Figure S1).

**Figure 1:**
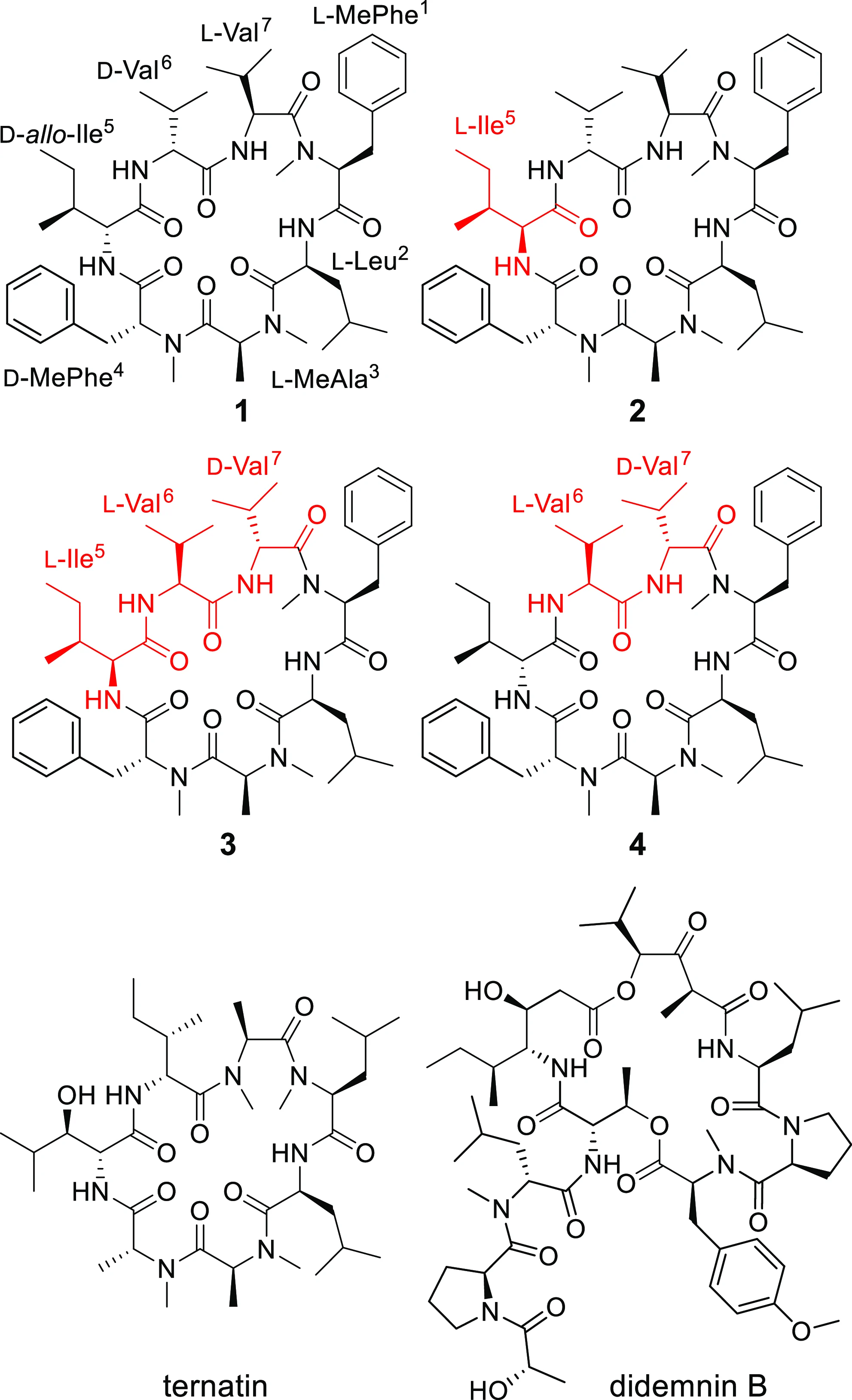
Molecular structures of paraisariamide A (**1**) and synthetic diastereomers: [l-lle^5^]-paraisariamide A (**2**), [l-lle^5^, l-Val^6^, d-Val^7^]-paraisariamide A (**3**), [l-Val^6^, d-Val^7^]-paraisariamide A (**4**), ternatin peptide, and didemnin B.

Access to pure synthetic diastereomers of **1** afforded the opportunity to investigate the potential influence of stereochemistry on the emerging structure–activity relationship (SAR) for the Psd series.^1^ Further, our initial investigation of the biological activity profile of Psd A (**1**), which has three *N*-methyl substituents rather than the four in Psds E–H,^1^ had revealed assay-specific activity for **1**. While only weakly cytotoxic to HCT116 colon cancer cells (CC_50_ = 10 μM), **1** showed moderate (nanomolar) activity in a second phenotypic assay that reports on the functional status of the eukaryotic secretory pathway.^1^ In these studies, human U87-MG glioblastoma cells expressing a secreted photoprotein, *Gaussia* luciferase (GLuc), were exposed to increasing concentrations of **1** (1–10 μM), ternatin (0.1 nM to 1 μM), structurally unrelated eEF1A inhibitor didemnin B (0.1 nM to 1 μM),^5,6^ or vehicle (0.1% DMSO) for 18 h before analysis of GLuc expression in the conditioned medium.^1,7^ Although Psd A (**1**) was a less potent inhibitor of secreted GLuc than ternatin or didemnin B, **1** displayed a clear concentration-dependent reduction in GLuc expression and was as efficacious as both control protein synthesis inhibitors in these experiments.^1^ However, in a side-by-side comparison with Psds E–H, **1** failed to inhibit expression of nascent AHA-labeled proteins in living HCT116 cells at the highest (3 μM) concentration tested (Figure S2).^1^

Given the high sensitivity of the GLuc assay for sensing compounds that decrease protein processing and stress in the endoplasmic reticulum (ER),^7^ we utilized this assay to compare the pharmacology of **1**–**4**. Using the same experimental design, GLuc-U87 cells were exposed to increasing concentrations of Psd A (**1**), diastereomers **2**–**4** (1–10 μM), ternatin (0.3 nM to 1 μM), didemnin B (0.1 nM to 1 μM), apratoxin A (300 nM), or vehicle (0.1% DMSO) for 18 h. Aliquots of the conditioned cell culture medium were then removed and assessed for the presence of GLuc activity. Psd A (**1**) and all three synthetic variants (**2**–**4**) induced concentration-dependent decreases in GLuc activity relative to vehicle-treated cells with similar nanomolar potency (Figure 2A and Table 1). Consistent with our earlier analysis,^1^ both control inhibitors of ribosomal protein synthesis, dideminin B and ternatin, were active in this assay (Figure 2A). Didemnin B was approximately 16-fold more potent than ternatin and at least 75-fold more potent than **1**–**4** as an inhibitor of extracellular GLuc expression (Table 1). Using apratoxin A (300 nM), a direct inhibitor of Sec61-dependent import into the conventional secretory pathway,^8–10^ as an independent measure of compound efficacy, we found that all Psds decreased GLuc expression by at least 60% of the control, with **1** and **3** approaching the efficacy observed at maximal concentrations of didemnin B, ternatin, and apratoxin A by 18 h (Figure 2A).

**Figure 2:**
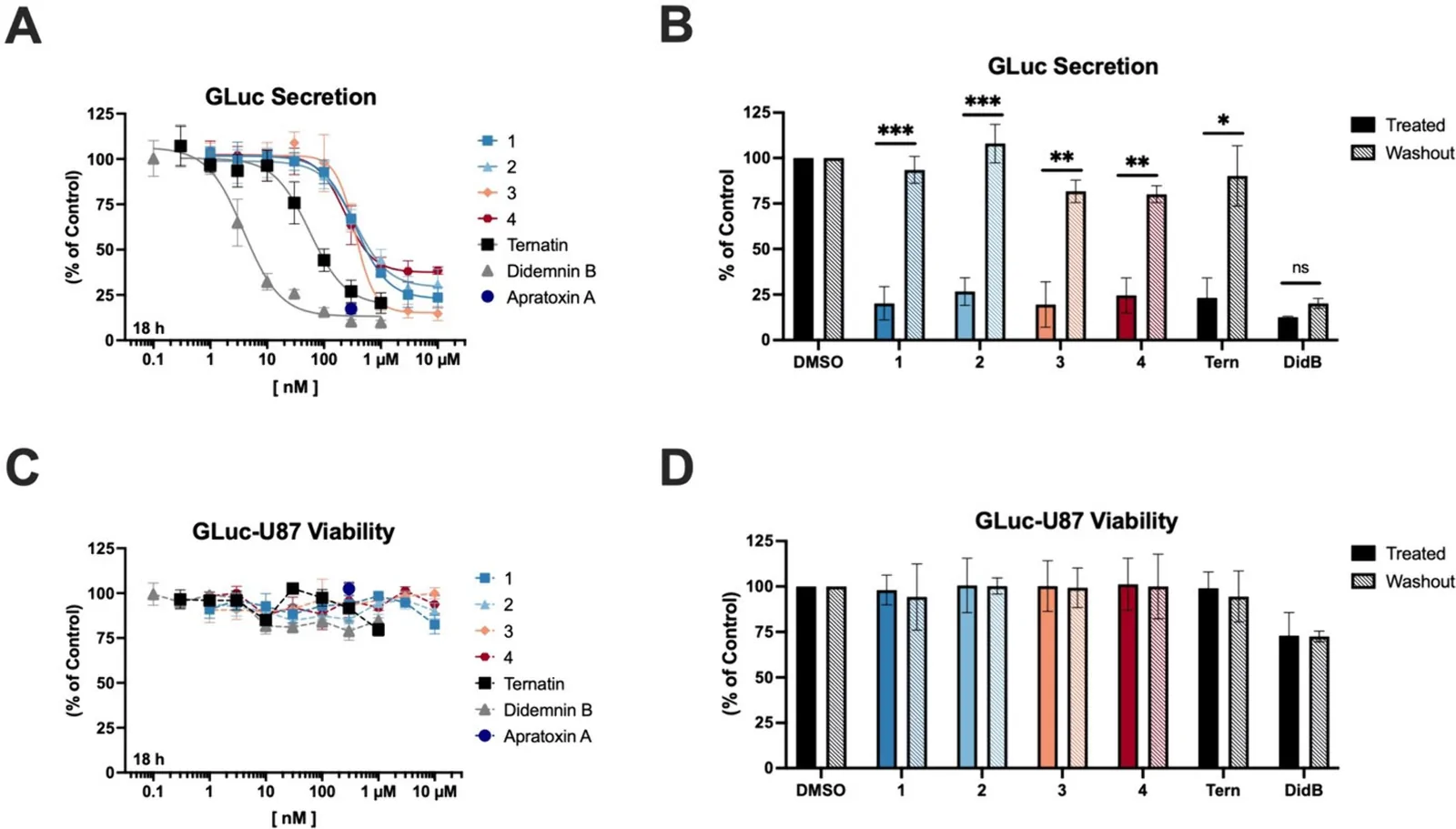
Comparative bioactivity of paraisariamide A (**1**) and synthetic diastereomers (**2**–**4**) relative to known elongation factor inhibitors, ternatin and didemnin B. Analysis of the secretory function of human U87-MG glioblastoma cells expressing the reporter *Gaussia* luciferase (GLuc). (A) GLuc expression was assessed in the conditioned medium 18 h after exposure to **1**–**4**, ternatin, didemnin B, apratoxin A, or vehicle (0.1% DMSO). (B) Cells were treated for 18 h and GLuc-expression-assessed 24 h after a rinse with PBS and replacement with fresh medium with (treatment) or without (washout) the compound. (C) Viability (18 h) of GLuc-expressing U87 cells in panel A and (D) viability (42 h) of GLuc-expressing U87 cells in panel B. Cell viability was measured by quantification of ATP at the end point. Data points represent the mean ± SE (*n* = 3 wells per treatment), expressed as a % of vehicle-treated cells, from a comparison that was repeated 3 times.

**Table 1:** Comparative Bioactivity of Paraisariamide A (**1**) and Synthetic Diastereomers (**2**–**4**) Relative to Ternatin and Didemnin B^a^

compound	18 h GLuc secretion IC_50_ (nM)	18 h viability GLuc-U87 CC_50_ (μM)	72 h viability U87-MG CC_50_ (μM)	72 h viability HCT116 CC_50_ (μM)
**1**	367 ± 36	>10	>10	10
**2**	297 ± 60	>10	>10	9.2 ± 0.24
**3**	340 ± 7	>10	>10	9.5 ± 0.23
**4**	269 ± 17	>10	>10	4.6 ± 0.14
ternatin	58.8 ± 11	>1	>1	0.024 ± 0.005
didemnin B	3.6 ± 0.3	>1	0.006 ± 0.001	0.0033 ± 0.001

a Values represent concentrations for 50% inhibition of GLuc expression (IC_50_ ± SE) or cell viability [cytotoxic concentration (CC_50_ ± SE)] relative to the vehicle control.

The potent cytotoxicity of didemnin B to human cancer cells has long been attributed to a rapid onset and an irreversible mechanism of action. The high membrane permeability of the didemnin B lariat depsipeptide scaffold is thought to facilitate rapid target engagement such that inhibition of protein synthesis tends to correlate very well with antiproliferative activity and becomes irreversible within hours of exposure.^6,11,12^ To understand the pharmacological properties of the Psds, we studied the reversibility of **1**–**4**, relative to ternatin and didemnin B. GLuc-U87 reporter cells were treated with or without (0.1% DMSO) a fixed concentration of Psds **1**–**4** (500 nM), ternatin (100 nM), or didemnin B (10 nM), calculated to exceed the IC_50_ for inhibition of secreted GLuc for each compound (Table 1). After 18 h, the cell culture medium on all cells was removed and the adherent monolayer was washed 3 times with phosphate-buffered saline (PBS) and then replaced with either culture medium alone (washout) or retreated with the compound at the same fixed concentration (treated) for a further 24 h. Analysis of the conditioned medium at the end of these experiments revealed a statistically significant rescue of GLuc activity in the cell culture medium after removal and washout for all cells treated with either **1**–**4** or ternatin, relative to retreated cells (Figure 2B). In contrast, GLuc activity was not restored after washout of cells treated with didemnin B (Figure 2B), consistent with the short time frame for the onset of action of didemnin B in whole cells.^11^ Decreases in the secretory function of the adherent GLuc-U87 cells were not due to a loss in cell viability after 18 h (Figure 2C) or 42 h (Figure 2D) exposures to **1**–**4** or ternatin. GLuc-U87 cells exposed to didemnin B also remained viable at 18 h (Figure 2C) but showed a 25% decrease in viability at 42 h even after removal of didemnin B and medium replacement, consistent with an irreversible mechanism of action for this compound (Figure 2D).

Given that **2**, **3**, and **4** retained the ability to decrease GLuc secretion in living cells (Figure 2A), we tested the potential influence of stereochemistry on cytotoxic potential. Based on our original characterization of the Psd family,^1^ HCT116 and wild-type U87-MG cells were treated with increasing concentrations of Psd A (**1**) (1–10 μM), diastereomers **2**–**4** (1–10 μM), ternatin (0.3 nM to 1 μM), didemnin B (0.1 nM to 1 μM), apratoxin A (300 nM), or vehicle (0.1% DMSO), and cell viability was assessed after 72 h. All three diastereomers retained weak (micromolar) cytotoxic activity against HCT116 cells that was either comparable (**2** and **3**) or better (**4**) than **1** (Figure 3A and Table 1). In contrast, U87-MG cells remained resistant to **1**–**4** (10 μM) and ternatin (1 μM) at the highest concentrations tested (Figure 3B and Table 1). HCT116 colon cancer cells were also sensitive to ternatin, didemnin B, and apratoxin A exposure (Figure 3A and Table 1), whereas wild-type U87 glioblastoma cells were only sensitive to didemnin B and the unrelated control cyclic depsipeptide, apratoxin A (Figure 3B and Table 1). Although the mechanistic basis for the cell-type-specific bioactivity of the Psds is not yet known, marked differential sensitivity of human cancer cells has previously been reported for ternatin.^4^ When the antiproliferative activity of ternatin was evaluated against a 21 human cancer cell line panel, representing different solid tumor and blood cancer types, IC_50_ values ranged from low nM to >30 μM.^4^ Whereas didemnin B appears to have broad antiproliferative and cytotoxic activity toward multiple cancer cell lines and has been shown to accumulate in intact cells.^13,14^ Thus, consistent with previous reports of a rapid onset and irreversible action, didemnin B was equipotent in both the functional GLuc secretory assay and cell viability assays (Table 1). In contrast, the Psds appear to have a selectivity profile more like that of ternatin;^1^ however, some differences were noted in the present study. In general, Psds **1**–**4** were at least 30–17-fold less toxic to HCT116 cells than the effective concentrations for inhibition of GLuc, whereas ternatin showed the opposite pattern; ternatin was approximately 2-fold more potent as a cytotoxin after 72 h than the IC_50_ for GLuc inhibition at 18 h (Table 1).

**Figure 3:**
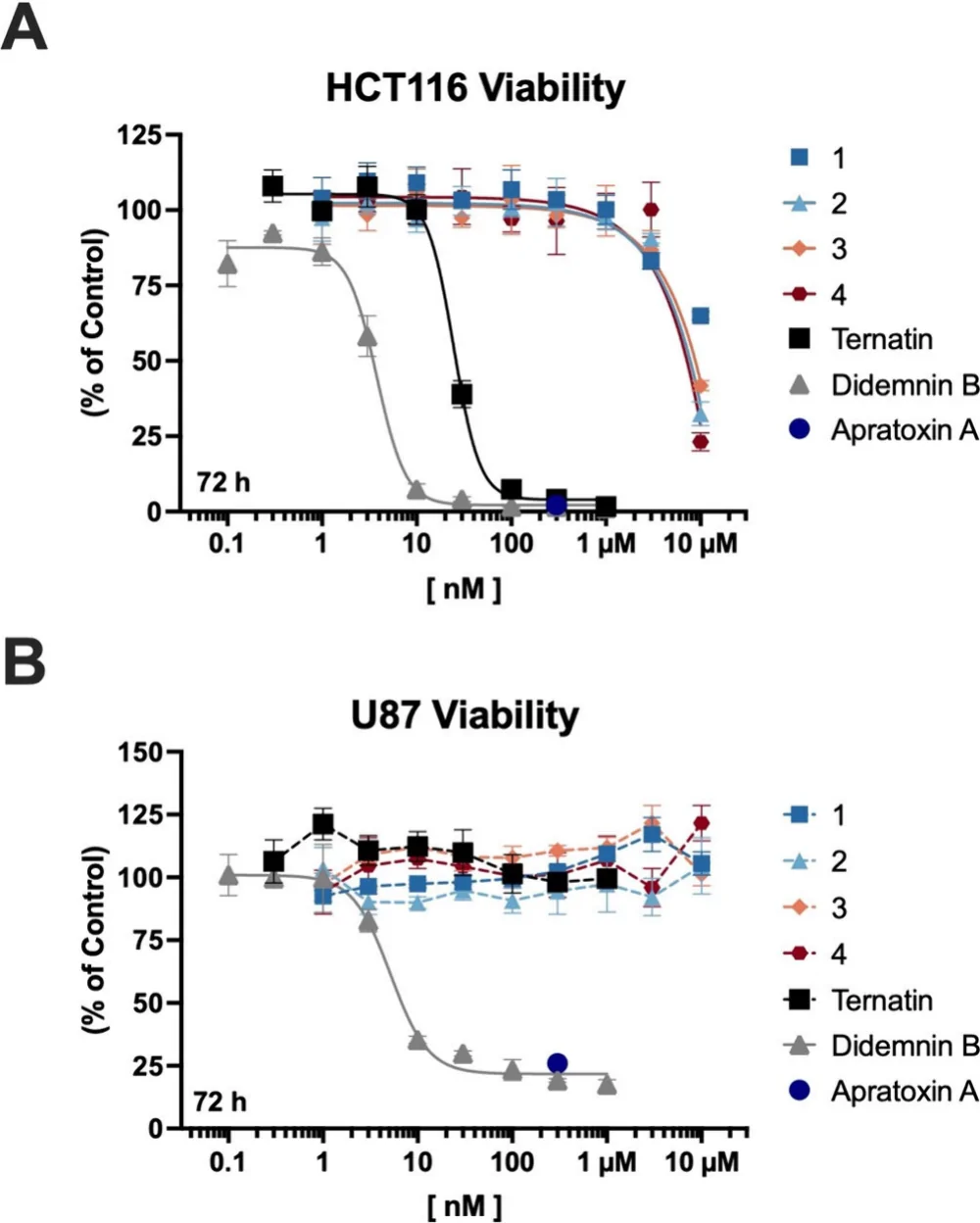
Comparative cytotoxicity of paraisariamide A (**1**) and synthetic diastereomers (**2**–**4**) relative to ternatin and didemnin B. Viability of (A) HCT116 colon cancer and (B) U87-MG glioblastoma cells at 72 h. Cells were exposed to increasing concentrations of the compound, apratoxin A (300 nM), or 0.1% DMSO, and viability was measured by quantification of ATP at the end point. Data points represent the mean ± SE (3 wells per treatment) from a comparison that was repeated at least 4 times.

The GLuc reporter assay can detect both site-specific inhibitors of protein biosynthesis and compounds that are direct or indirect inducers of endoplasmic reticulum (ER) stress in the secretory pathway.^1,7,15–17^ To better understand the functional significance of Psd-induced decreases in GLuc secretion by these fully efficacious (Figure 2A and Table 1), less toxic variants (PsdA **1**–**4**), we used a form of the surface sensing of translation (SUnSET) assay that can detect global inhibitors of protein synthesis.^18,19^ The SUnSET assay is based on detection of nascent puromycilated proteins incorporated during the elongation stage of protein biogenesis and can be adapted for detection by western blot using antipuromycin antibodies.^18,19^ Preliminary studies with the control eEF1A inhibitor ternatin (0.3 and 1.0 μM) revealed no upregulation in expression of the ER chaperone glucose-regulated protein of 78 kDa (GRP78) in HCT116 cells after 4 h of treatment (Figure S3). GRP78 is a sensitive biomarker of the unfolded protein response that is rapidly induced in response to ER stress,^20^ and thus, these results suggested that translation of GRP78 is sensitive to ternatin and eventually compromises the ability of the cell to activate a typical ER stress response. We also evaluated expression of eEF1A in HCT116 cell lysates after short-term exposure to ternatin and found eEF1A levels to be stable under these experimental conditions (Figure S3), whereas a more potent synthetic analogue of ternatin (ternatin 4) has been shown previously to trigger proteasomal degradation of eEF1A and related translation factors.^4,21^

For analysis of global protein synthesis, HCT116 cells were cultured in the presence and absence of Psd A **1**–**4** (10 μM), ternatin (1.0 μM), or cycloheximide (25 μM) for 60 min under standard growth conditions. A pulse of puromycin (5 μg/mL) was added to the medium of all vehicle- and compound-treated plates 15 min before the assay end point when whole-cell lysates were collected and normalized for protein concentration. Immunoblot analysis of puromycilated proteins revealed a statistically significant decrease in the nascent protein in the presence of **1**, **3**, ternatin, and cycloheximide, whereas any change in response to **2** and **4** was not statistically significant (Figure 4A and B). No statistically significant change in GRP78 or eEF1A expression was seen following exposure to **1**–**4**, although expression levels of GRP78 protein showed some variation in response to ternatin and cycloheximide in these studies (Figure 4B). Together these results demonstrate the feasibility of using the SUnSET technique as a non-radioactive method to survey the potential of natural products to induce global inhibition of protein synthesis. In the present study, inhibition of total protein synthesis by the control fungal peptide ternatin was qualitatively indistinguishable from the action of cycloheximide (Figure S4 and Figure 4). When studied relative to these well-characterized positive controls, we demonstrate that short-term (1 h) exposure to Psd A (**1**) and at least [l-lle^5^, l-Val^6^, d-Val^7^]-paraisariamide A (**3**) results in suppression of global protein synthesis, albeit with weaker biological activity than the more toxic members of the Psd series.^1^

**Figure 4:**
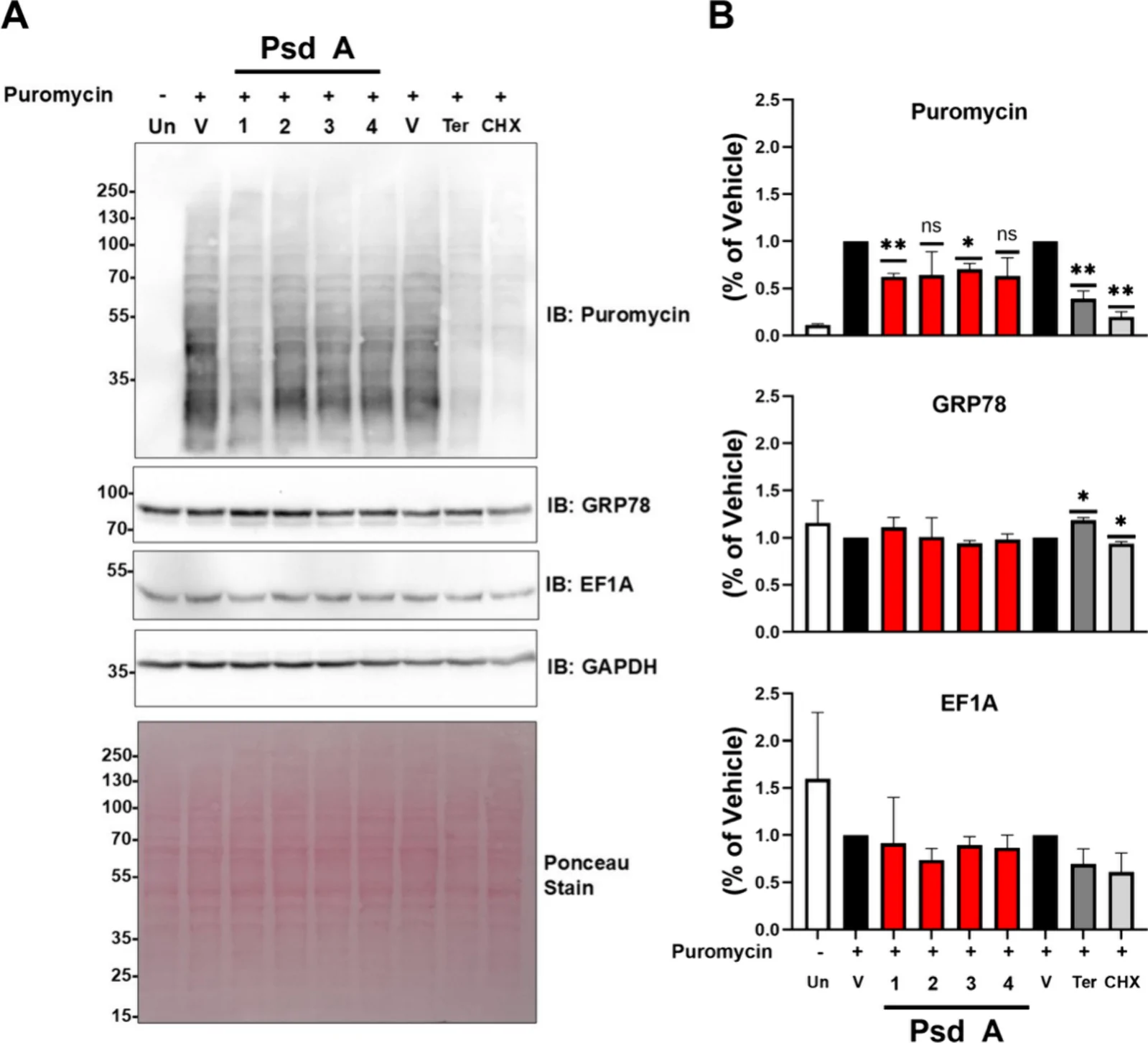
SUnSET analysis of nascent protein synthesis in the presence and absence of PsdAs **1**–**4**, ternatin, or cycloheximide. HCT116 colon cancer cells were cultured in 6-well plates and were either left untreated (Un) or exposed to solvent vehicle (V, 0.1% DMSO), PsdA **1** (10 μM), synthetic diastereomers **2** (10 μM), **3** (10 μM), or **4** (10 μM), ternatin (Ter, 1.0 μM), or cycloheximide (CHX, 25 μM) for 1 h with a pulse (+) of puromycin (5 μg/mL) added 15 min before collection of whole-cell lysates. (A) Immunoblot analysis of puromycilated proteins GRP78 and eEF1A expression relative to a loading control (GAPDH) and Ponceau stain of the whole PVDF membrane prior to immunoblot analysis of puromycin expression. (B) Quantification of immunoblot data from three independent experiments. Statistical significance of change relative to the vehicle (0.1% DMSO) with puromycin chase (+) is (∗) *p* < 0.05 and (∗∗) *p* < 0.01.

In summary, we demonstrate that PsdA (**1**), the founding member of a new family of cyclic heptapeptide natural products from the *Paraisaria* genus of insect-pathogenic fungi, is a reversible inhibitor of total protein synthesis with a ternatin-like cell-line selectivity profile. Further, several synthetic diastereomers of **1**, [l-lle]^5^-paraisariamide A (**2**), [l-lle^5^, l-Val^6^, d-Val^7^]-paraisariamide A (**3**), and [l-Val^6^, d-Val^7^]-paraisariamide A (**4**), retained molecular conformations that were sufficient for pharmacological activity. Like ternatin, all structural variants of **1** induced non-lethal stress in U87-MG cells; these compounds inhibited the expression of a secreted reporter (*Gaussia* luciferase) in living cells with nanomolar potency and in a reversible manner. We observed no upregulation of the ER stress biomarker GRP78 after short-term exposure; however, all variants of **1** eventually showed weak cytotoxic activity toward HCT116 colon cancer cells after 72 h. Natural product inhibitors of protein biosynthesis have been discovered from numerous ecological niches and continue to serve as important tool compounds with drug-like characteristics.^22,23^ Although the direct binding target of the Psds has yet to be determined, the discovery of less toxic inhibitors of protein synthesis based on the Psd chemotype is anticipated to inform future SAR studies of these ternatin-like molecules.

## Experimental Procedures

### Chemicals, Reagents, and Antibodies

Psd A (**1**) was synthesized as described previously.^1^ No unexpected or unusually high safety hazards were encountered in the synthesis of diastereomers **2**–**4**. Apratoxin A was from a laboratory culture of Red Sea cyanobacteria.^24^ The purity of non-commercial compounds was determined to be >95% by HPLC analysis and were supplied in dry form, reconstituted in 100% cell-culture-grade DMSO, Millipore Sigma (Burlington, MA), aliquoted, and stored as concentrated (10 mM) stock solutions in amber borosilicate glass vials at −20 °C for use in biological studies. Ternatin (peptide) was from Cayman Chemical (Ann Arbor, MI); cycloheximide was from Millipore Sigma; puromycin was from Thermo Fisher Scientific, Inc. (Waltham, MA); and coelenterazine was from Promega Corporation (Madison, WI). The antipuromycin antibody (clone 12D10) was from Millipore Sigma; the anti-GAPDH antibody (60004-1-g) was from Proteintech (Rosemont, IL); and eEF1A (2551) and GRP78 (BiP, 3177) primary antibodies plus all HRP-conjugated secondary antibodies were from Cell Signaling Technology, Inc. (Danvers, MA). General laboratory reagents were from VWR International (Radnor, PA).

### Mammalian Cell Culture

Human U87-MG glioblastoma and HCT116 colon cancer cells were from the American Type Culture Collection (ATCC, Manasses, VA). Generation of the reporter cell line (GLuc-U87) from wild-type U87-MG cells has been described previously.^7^ GLuc-U87 and U87-MG glioblastoma cells were maintained in minimum essential medium (MEM) with Earl’s salts and l-glutamine (Corning Life Sciences), supplemented with 10% fetal bovine serum (FBS, Hyclone, Logan, UT) and 1% penicillin/streptomycin. HCCT116 cells were maintained in McCoy’s 5A medium supplemented with 10% FBS and 1% penicillin/streptomycin. Cells were maintained at 37 °C in an atmosphere of 5% CO_2_.

### *Gaussia* Luciferase (GLuc) Secretory Assay

GLuc-U87 cells were seeded at 3000 cells/well into 96-well plates and allowed to adhere overnight. The next day, seeding medium was replaced with complete medium containing test compounds or vehicle (0.1% DMSO), and plates were returned to the incubator. At the end point, conditioned cell culture medium (20 μL) was removed from each well and transferred to 96-well white-walled plates. Expression of GLuc was assessed by the addition of substrate coelenterazine into each well (final concentration of 1.2 μM), and luminescent signals were measured using a multimode microplate reader (Biotek Synergy HT) with Gen5 software. For analysis of reversibility, GLuc-U87 cells were treated for 18 h, the cell culture medium was then removed, and the cell monolayer was gently rinsed with phosphate-buffered saline (3× PBS). The medium in all wells was then replaced with fresh complete medium in the presence or absence (medium only) of the compound or vehicle (consistent with the original treatment), and all plates returned to the incubator for a further 24 h. GLuc expression was assessed at the extended (42 h) end point as described above.

### Cell Viability Assays

Wild-type U87-MG or HCT116 cells were seeded into standard 96-well plates at a density of 3000 cells/well and allowed to adhere overnight before treatment with test compounds or vehicle (0.1% DMSO). The viability of all cells, including adherent GLuc-U87 cells used for monitoring cell secretion, was assessed at the end point of each assay using a CellTiter-Glo luminescent cell viability assay designed to detect ATP in metabolically active cells (Promega Corp., Madison, WI). Luminescent signals were measured using the same Biotek Synergy HT multimode microplate reader.

### Surface Sensing of Translation (SUnSET) Assay and Western Blot Analysis

All SUnSET assays followed the protocol established by Piecyk and co-authors,^19^ adapted from an original method by Schmidt and co-authors.^18^ Briefly, HCT116 cells were seeded at 200 000 cells/well into 6-well plates and allowed to adhere overnight. The following day, cells were treated with the test compound or vehicle (0.1% DMSO) for 1 h. At 15 min before the end of the treatment, puromycin, which is structurally similar to tyrosyl-tRNA, was added to each treated well to a final concentration of 5 μg/mL; untreated cells received no puromycin. Cell lysates were collected immediately in fresh ice-cold lysis buffer containing 50 nM Tris–HCl (PH 7.5), 1 mM EDTA, 1 mM EGTA, 1% Triton X-100, 0.27 M sucrose, 50 mM sodium fluoride, 1 mM sodium orthovanadate, 5 mM sodium pyrophosphate, 1 mM PMSF, and 1 mM benzamidine. All cell lysates were cleared by centrifugation (16000*g* for 20 min at 4 °C), and the protein concentration was determined by the bicinchoninic acid (BCA) method (Thermo Fisher Scientific, Waltham, MA).

For immunoblot analysis, equal concentrations of cell lysate were separated by SDS–PAGE gel. Proteins were then transferred onto PVDF membranes (Thermo Fisher Scientific) in a transfer buffer containing 25 nM Tris, 192 mM glycine, and 20% methanol. The total protein was always visualized using Ponceau S stain (VWR International, Radnor, PA). Membranes were then blocked in 5% (m/v) non-fat milk in 50 mM Tris–HCl at pH 7.4 and 150 mM NaCl (TBS) with 0.05% Tween 20 (TBS-T) and incubated at 4 °C overnight with appropriate primary antibodies in 5% (w/v) bovine serum albumin (BSA) in TBS-T. The following day, membranes were washed with TBS-T for 3 × 5 min and incubated with the appropriate HRP-conjugated secondary antibodies for 1 h at RT. Membranes were washed in TBS-T (3 × 5 min at RT), and proteins were detected by chemiluminescence (Amersham ECL, GE Healthcare, Chicago, IL, U.S.A.), using an iBright Imager system (Thermo Fisher Scientific).

### Data Analysis

Concentration–response relationships were analyzed by a nonlinear regression analysis fit to a logistic equation using GraphPad Prism Software (GraphPad Software, Inc., San Diego, CA). For SUnSET and immunoblot studies, signals were normalized to the intensity of GAPDH and quantified using ImageJ software (rsbweb.nih.gov/ij). Statistical significance of data was assessed using a one-way analysis of variance (ANOVA) followed by a Student’s *t* test. *p* values less than 0.05 were considered significant.

## Supplementary Material



## Notes

Experimental procedures for chemical synthesis, chemical characterization of new synthetic peptides, and supporting biological figures (PDF)

## Abbreviations Used

AHA: azidohomoalanine
CHX: cycloheximide
DMSO: dimethyl sulfoxide
EDTA: ethylenediaminetetraacetic acid
EGTA: ethylene glycol tetraacetic acid
ER: endoplasmic reticulum
eEF1A: eukaryotic elongation factor 1A
Ile: isoleucine
GAPDH: glyceraldehyde 3-phosphate dehydrogenase
GLuc: *Gaussia* luciferase
GRP78: glucose-regulated protein of 78 kDa
HRP: horseradish peroxidase
Leu: leucine
MeAla: methylalanine
MePhe: methylphenylalanine
SAR: structure–activity relationship
SDS–PAGE: sodium dodecyl sulfate polyacrylamide gel electrophoresis
SUnSET: surface sensing of translation
TAMRA: tetramethylrhodamine
Val: valine
