# Supplementary material for: Synthetic Diastereomers of the Fungal Cycloheptapeptide Paraisariamide A Retain Ternatin-Like Biological Activity
Source: ACS Med Chem Lett. 2026 Jul 1;17(8):1787–94. doi: 10.1021/acsmedchemlett.6c00180 (PMC13488079; doi:10.1021/acsmedchemlett.6c00180)
Supplement: Supplementary file 1 [file ml6c00180_si_001.pdf]

Supporting Information

Synthetic diastereomers of the fungal cycloheptapeptide Paraisariamide A retain ternatin-like biological activity

Sophia E. Bonar [1,#], Daphne R. Mattos [1,#], Takumi Arai [2], Yui Tagami [2], Dean A. Buch[1], Richard M. Tehan[1], Kerry L. McPhail [3], Shinya Oishi [2*] and Jane E. Ishmael [1*]

[1] Department of Pharmaceutical Sciences, College of Pharmacy, Oregon State University, Corvallis, Oregon 97331, USA

[2] Laboratory of Medicinal Chemistry, Kyoto Pharmaceutical University, Kyoto 607-8412, Japan

[3] Department of Medicinal Chemistry, College of Pharmacy, University of Utah, Salt Lake City, Utah, 84103, USA

Email: jane.ishmael@oregonstate.edu and soishi@mb.kyoto-phu.ac.jp

Table of Contents

Page

S3 General Experimental Procedures for Chemical Synthesis

S5 Scheme S1:Synthesis of [L-Ile^{5}, L-Val^{6}, D-Val^{7}]-paraisariamide A (**3**) and [L-Val^{6}, D-Val^{7}] -paraisariamide A (**4**)

S6 Scheme S2: Synthesis of [L-Ile^{5}]-paraisariamide A (**2**) via an alternative synthetic route.

S7 Figure S1: Analytical HPLC chromatograms of newly synthesized peptides

S8 Figure S2: Comparison of inhibition of nascent protein synthesis by cycloheximide and synthetic Psds A, E, F, G and H.

S9 Figure S3: Immunoblot analysis of GRP78 and eEF1A expression in HCT116 colon cancer cells.

S10 Figure S4: SUnSET assay showing inhibition of nascent protein synthesis by ternatin and cycloheximide.

S11 References

General Experimental Procedures for Chemical Synthesis

For analytical HPLC, a Cosmosil 5C18-ARII column (4.6 × 250 mm, Nacalai Tesque, Inc.) was employed with a linear gradient of CH_3CN in H_2O (with 0.05% TFA), and eluting products were detected by UV at 220 nm. Preparative HPLC was performed using a Cosmosil 5C18-ARII preparative column (20 × 250 mm, Nacalai Tesque, Inc.). The compound purity of peptides **1**–**4** was determined to be >95% by HPLC analysis. No unexpected or unusually high safety hazards were encountered. Synthesis of peptide **1** (natural paraisariamide A) was previously reported. [1]

General Procedure for Solid-Phase Peptide Synthesis.

Deprotection of Fmoc group. The Fmoc-protected peptidyl resin was treated with 20% piperidine/DMF for 20 min.

Coupling reaction of Fmoc-protected amino acid using DIC/HOBt. DIC (23 μL, 0.15 mmol) was added to a solution of Fmoc amino acid (0.15 mmol) and HOBt·H_2O (23 mg, 0.15 mmol) in DMF. The whole was poured into the peptidyl resin (0.050 mmol), and the reaction was continued for 1.5 h at 40 °C.

Coupling reaction of Fmoc-protected amino acid using DIC/HOAt. DIC (23 μL, 0.15 mmol) was added to a solution of Fmoc amino acid (0.15 mmol) and HOAt (20 mg, 0.15 mmol) in DMF. The whole was poured into the peptidyl resin (0.100 mmol), and the reaction was continued for 1.5 h at 40 °C.

Cleavage from the resin. The peptidyl resin was treated with 1,1,1,3,3,3-hexafluoropropan-2-ol (HFIP)/CH_2Cl_2 (3:7) for 2 h at room temperature. After filtration of the residual resin, the filtrate was concentrated under reduced pressure to give a crude peptide, which was used for the next step without further purification.

[L-Ile^5]-paraisariamide A (2). The linear peptide was constructed by solid-phase peptide synthesis on H-Leu-(2-Cl)Trt resin 13 (0.75 mmol/g, 67 mg, 0.050 mmol). After the cleavage from the resin by the standard protocol, EDCI·HCl (29.2 mg, 0.152 mmol) was added to a

solution of linear peptide (12.9 mg), HOAt (20.7 mg, 0.152 mmol), and DIEA (106 µL, 0.609 mmol) in dry DMF (15.2 mL) at 0 °C. The reaction mixture was allowed to warm up to room temperature and the stirring was continued for 13.5 h. The reaction mixture was concentrated and the residue was purified by HPLC (65–95% linear gradient of CH_3CN in H_2O with 0.05% (v/v) TFA at 60 °C) to provide [L-Ile^5]-paraisariamide A (**2**) (4.2 mg, 10% from resin).

Peptide **2**: $[\alpha]^{23}_D$ +8.5 (*c* 0.24, $CHCl_3$) HRMS (FAB) calcd for $C_{46}H_{70}O_7N_7$ $[M+H]^+$: 832.5337; found: 832.5331.

[L-Ile^5, L-Val^6, D-Val^7]-paraisariamide A (3) and [L-Val^6, D-Val^7]-paraisariamide A (4). The linear peptide was constructed by solid-phase peptide synthesis on Fmoc-D-*allo*-Ile-(2-Cl)Trt resin (0.661 mmol/g, 76 mg, 0.050 mmol). After the cleavage from the resin by the standard protocol, EDCI·HCl (103.9 mg, 0.542 mmol) was added to a solution of linear peptide (46.1 mg), HOAt (73.8 mg, 0.542 mmol), and DIEA (377.8 µL, 2.17 mmol) in dry DMF (50 mL) at 0 °C. The reaction mixture was allowed to warm up to room temperature and the stirring was continued overnight. The reaction mixture was concentrated and the residue was purified by HPLC to provide [L-Ile^5, L-Val^6, D-Val^7] -paraisariamide A (**3**) (3.6 mg, 8.7% from resin) and [L-Val^6, D-Val^7]-paraisariamide A (**4**) (14.1 mg, 33% from resin).

Peptide **3**: $[\alpha]^{23}_D$ –68.2 (*c* 0.41, $CHCl_3$) HRMS (FAB) calcd for $C_{46}H_{69}O_7N_7Na$ $[M+Na]^+$: 854.5156; found: 854.5176.

Peptide **4**: $[\alpha]^{23}_D$ –48.1 (*c* 1.0, $CHCl_3$) HRMS (FAB) calcd for $C_{46}H_{70}O_7N_7$ $[M+H]^+$: 832.5337; found: 832.5321.

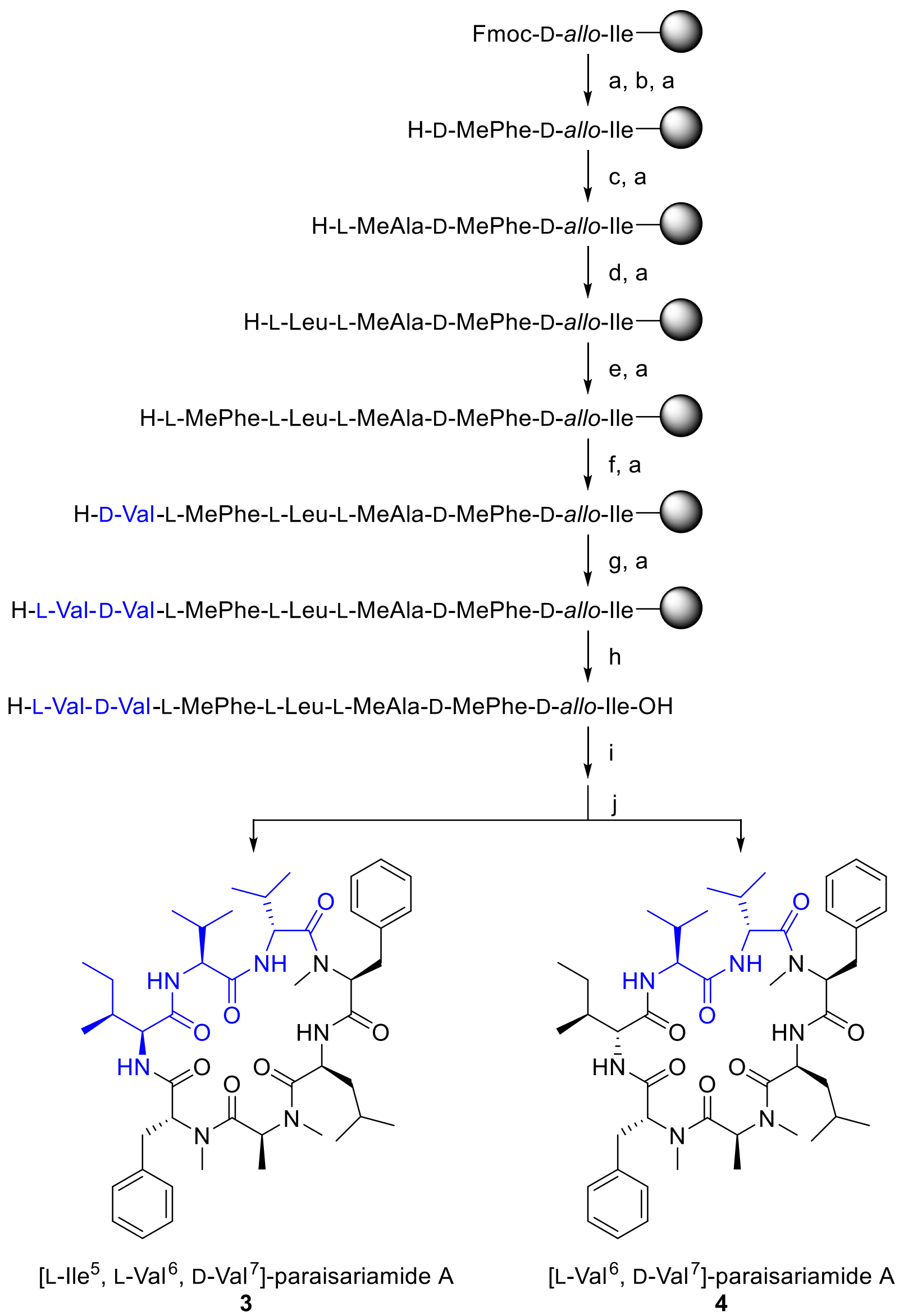

Scheme S1. Synthesis of [L-Ile^5, L-Val^6, D-Val^7]-paraisariamide A (**3**) and [L-Val^6, D-Val^7]-paraisariamide A (**4**).

Reagent and conditions: (a) 20% piperidine/DMF; (b) Fmoc-D-MePhe-OH, DIC, HOBt, DMF; (c) Fmoc-MeAla-OH, DIC, HOAt, DMF, 40 °C; (d) Fmoc-Leu-OH, DIC, HOAt, DMF, 40 °C; (e) Fmoc-MePhe-OH, DIC, HOBt, DMF; (f) Fmoc-D-Val-OH, DIC, HOAt, DMF, 40 °C; (g) Fmoc-Val-OH, DIC, HOBt, DMF; (h) 30% HFIP/CH_2Cl_2; (i) EDCI·HCl, HOAt, DIEA, DMF, 0 °C to rt.; (j) HPLC separation.

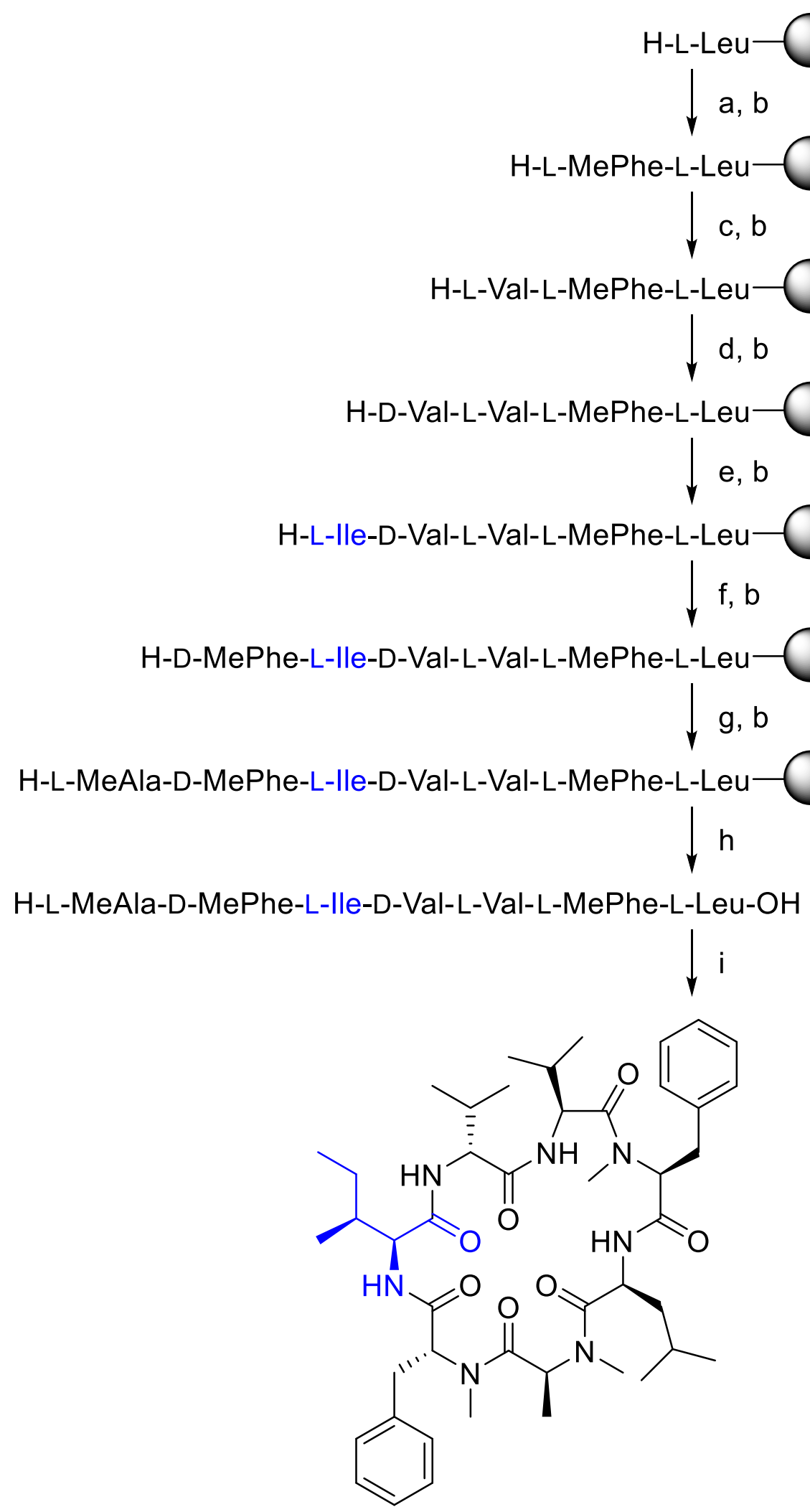

Scheme S2. Synthesis of [L-Ile^{5}]-paraisariamide A (**2**) via an alternative synthetic route.

Reagent and conditions: (a) Fmoc-MePhe-OH, DIC, HOBt, DMF; (b) 20% piperidine/DMF; (c) Fmoc-Val-OH, DIC, HOAt, DMF, 40 °C; (d) Fmoc-D-Val-OH, DIEA, HATU, DMF; (e) Fmoc-Ile-OH, DIEA, HATU, DMF; (f) Fmoc-D-MePhe-OH, DIEA, HATU, DMF; 40 °C (g) Fmoc-MeAla-OH, DIC, HOAt, DMF, 40 °C; (h) 30% HFIP/CH_2Cl_2; (i) EDCI·HCl, HOAt, DIEA, DMF, 0 °C to rt.

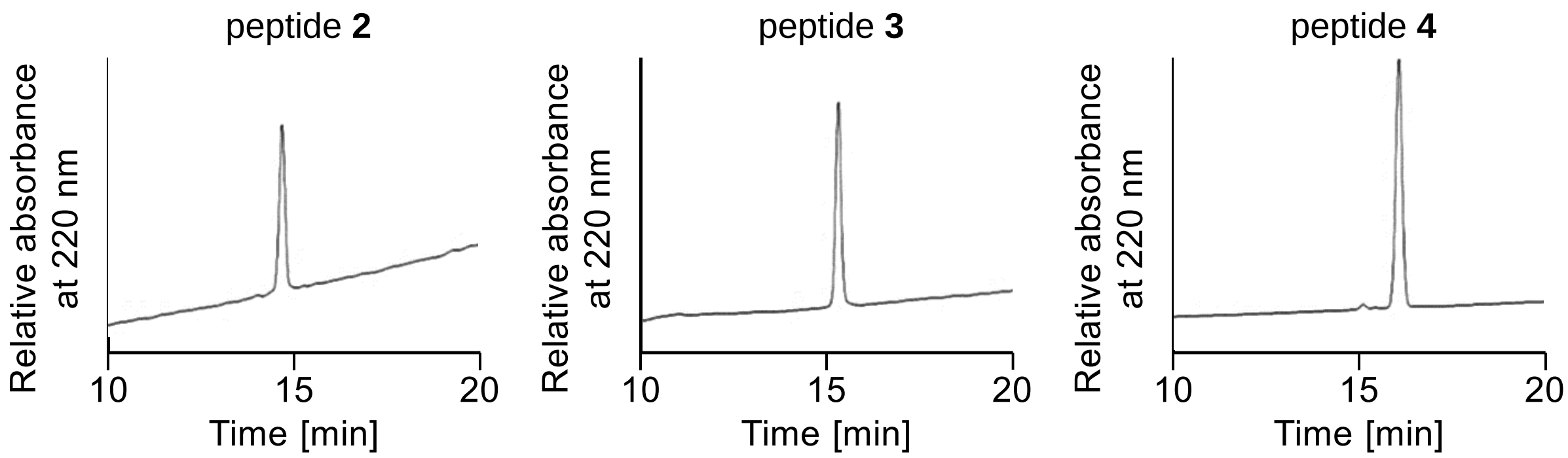

Figure S1: Analytical HPLC chromatograms of newly synthesized peptides

HPLC conditions: Cosmosil 5C18-ARII column (Nacalai Tesque, 4.6 × 250 mm). Linear gradient of 60–80% CH_3CN containing 0.05% TFA at a flow rate of 1 mL/min over 20 min at 60 °C.

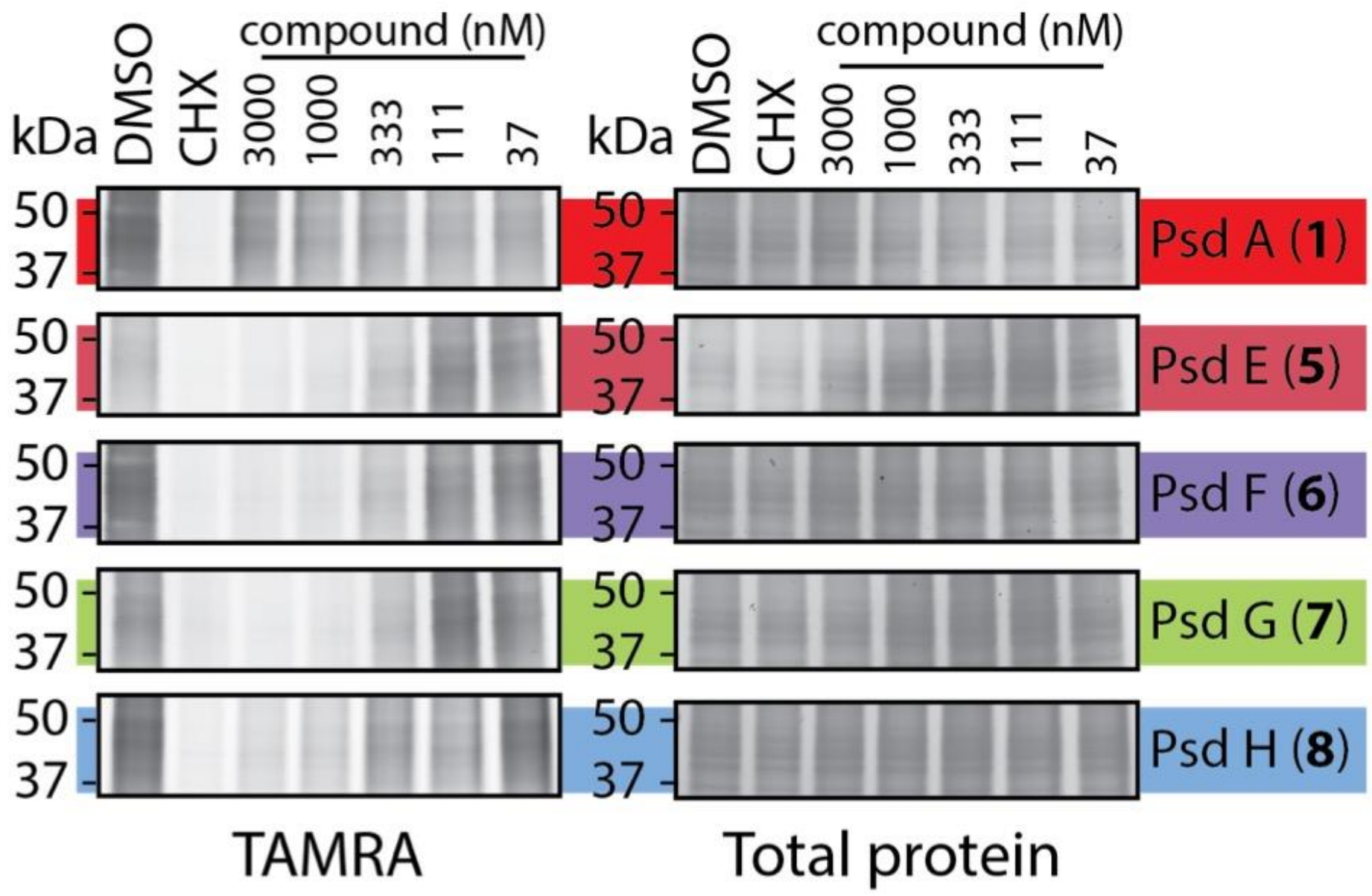

Figure S2. Comparison of inhibition of nascent protein synthesis by cycloheximide and synthetic Psd A (1), Psd E (5), Psd F (6), Psd G (7) and Psd H (8).

HCT-116 cells were labeled with L-AzidoHomoAlanine (L-AHA) in the presence of increasing concentrations (nM) of Psds (37 – 3000 nM), cycloheximide (CHX), or 0.1% DMSO. (Left) Newly synthesized proteins were detected by installation of a TAMRA fluorophore to L-AHA via click chemistry. (Right) Total protein for each treatment. Adapted from. [1]

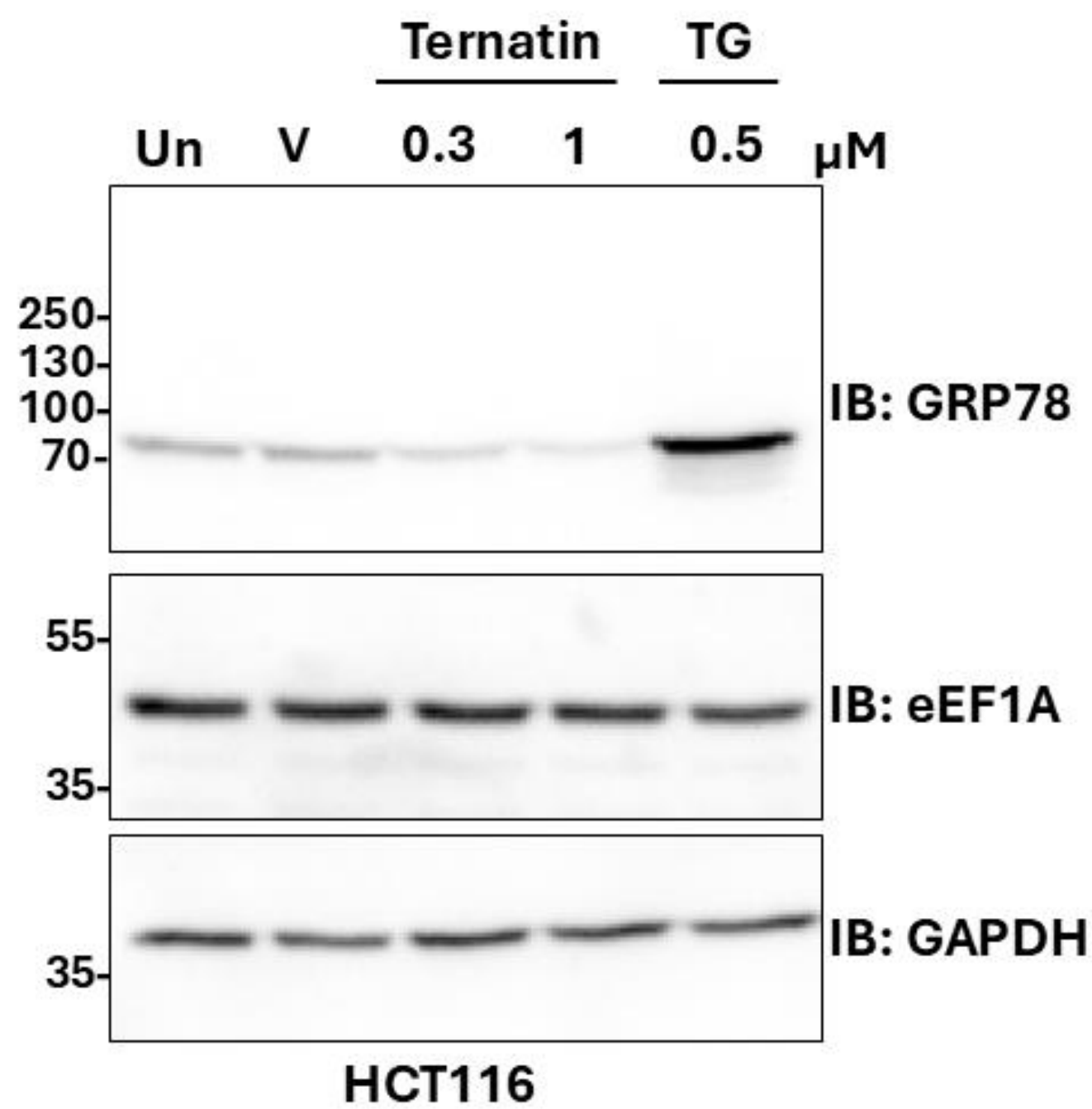

Figure S3. Immunoblot analysis of GRP78 and eEF1A expression in HCT116 colon cancer cells.

HCT116 cells were cultured in 6-well plates and were either untreated (Un) or exposed to solvent vehicle (V; 0.1% DMSO), ternatin (0.3 μM and 1.0 μM) or thapsigargin (TG; 0.5 μM) for 4 h. Images show representative expression of GRP78 and eEF1A relative to a loading control (GAPDH).

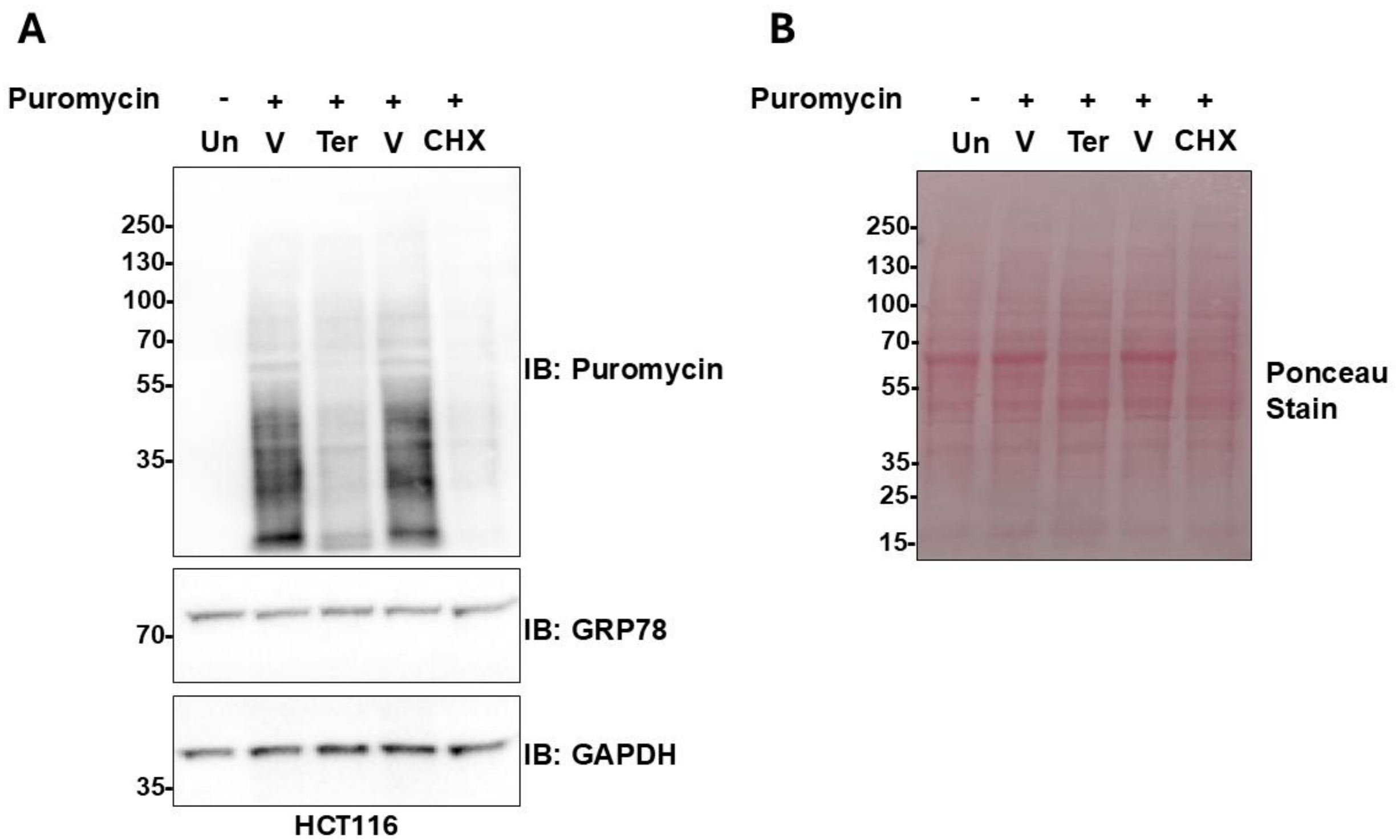

Figure S4. SUnSET assay showing inhibition of nascent protein synthesis by ternatin and cycloheximide.

HCT116 cells colon were cultured in 6-well plates and were either untreated (Un) or exposed to solvent vehicle (V; 0.1% DMSO), ternatin (Ter; 1.0 µM) or cycloheximide (CHX; 25 µM) for 1h and exposed (+) to puromycin (5 µg/mL) 15 min before collection of whole-cell protein lysates. [2,3] (A) Immunoblot analysis of puromycilated protein and GRP78 expression relative to a loading control (GAPDH) after a 15 min pulse. (B) Ponceau stain of whole PVDF membrane prior to immunoblot analysis of puromycin expression.

References

1. Tehan, R. M.; Mattos, D. R.; Arai, T.; Suzuki, R.; Paatero, A. O.; Tranter, D.; Dooley, C. B.; Rodriguez, G. J.; Spatafora, J. W.; Paavilainen, V. O.; Oishi, S.; Ishmael, J. E.; McPhail, K. L., Paraisariamides: Cycloheptapeptide Toxins from Entomopathogenic Fungi (Paraisaria spp.) That Inhibit Total Protein Synthesis. *J. Nat. Prod.* **2026**, *89* (1), 82-94.

2. Piecyk, M.; Fauvre, J.; Duret, C.; Chaveroux, C.; Ferraro-Peyret, C., SUrface SEnsing of Translation (SUnSET), a Method Based on Western Blot Assessing Protein Synthesis Rates in vitro. *Bio. Protoc.* **2024**, *14* (3), e4933.

3. Schmidt, E. K.; Clavarino, G.; Ceppi, M.; Pierre, P., SUnSET, a nonradioactive method to monitor protein synthesis. *Nat. Methods* **2009**, *6* (4), 275-277.
